# Maternal mortality audit in Suriname between 2010 and 2014, a reproductive age mortality survey

**DOI:** 10.1186/s12884-017-1466-6

**Published:** 2017-08-29

**Authors:** Lachmi R. Kodan, Kim J. C. Verschueren, Jos van Roosmalen, Humphrey H. H. Kanhai, Kitty W. M. Bloemenkamp

**Affiliations:** 1 Department of Obstetrics, Academic Hospital Paramaribo (AZP), Paramaribo, Suriname; 2 Department of Obstetrics, Birth Centre Wilhelmina’s Children Hospital, University Medical Centre Utrecht, Utrecht, the Netherlands; 3 Athena Institute, VU University Amsterdam, Amsterdam, the Netherlands; 4 Department of Obstetrics, Leiden University Medical Centre, Leiden, the Netherlands; 5 Anton de Kom University, Paramaribo, Suriname

**Keywords:** Maternal mortality, Middle-income country, Mmr, Suriname, RAMoS, Underreporting

## Abstract

**Background:**

The fifth Millennium Development Goal (MDG-5) aimed to improve maternal health, targeting a maternal mortality ratio (MMR) reduction of 75% between 1990 and 2015. The objective of this study was to identify all maternal deaths in Suriname, determine the extent of underreporting, estimate the reduction, audit the maternal deaths and assess underlying causes and substandard care factors.

**Methods:**

A reproductive age mortality survey was conducted in Suriname (South-American upper-middle income country) between 2010 and 2014 to identify all maternal deaths in the country. MMR was compared to vital statistics and a previous confidential enquiry from 1991 to 1993 with a MMR 226. A maternal mortality committee audited the maternal deaths and identified underlying causes and substandard care factors.

**Results:**

In the study period 65 maternal deaths were identified in 50,051 live births, indicating a MMR of 130 per 100.000 live births and implicating a 42% reduction of maternal deaths in the past 25 years. Vital registration indicated a MMR of 96, which marks underreporting of 26%. Maternal deaths mostly occurred in the urban hospitals (84%) and the causes were classified as direct (63%), indirect (32%) or unspecified (5%). Major underlying causes were obstetric and non-obstetric sepsis (27%) and haemorrhage (20%). Substandard care factors (95%) were mostly health professional related (80%) due to delay in diagnosis (59%), delay or wrong treatment (78%) or inadequate monitoring (59%). Substandard care factors most likely led to death in 47% of the cases.

**Conclusion:**

Despite the reduction in maternal mortality, Suriname did not reach MDG-5 in 2015. Steps to reach the Sustainable Development Goal in 2030 (MMR ≤ 70 per 100.000 live births) and eliminate preventable deaths include improving data surveillance, installing a maternal death review committee, and implementing national guidelines for prevention and management of major complications of pregnancy, childbirth and puerperium.

## Background

Reducing maternal mortality is one of the major challenges to health systems worldwide. United Nations’ Millennium Development Goal 5 (MDG-5) called for a 75% reduction of the maternal mortality ratio (MMR) between 1990 and 2015. The global MMR fell from 385 deaths per 100.000 live births in 1990 to 216 in 2015, corresponding to a decline of 44%. A vision of ending all preventable maternal deaths has emerged in 2015, being one of the Sustainable Development Goals (SDGs); it aims to reduce the global MMR to less than 70 deaths per 100.000 live births by 2030. Achievement of this target will require robust information systems with high-quality data, specifically on causes of death, as it is of great importance in informing decision-makers and ultimately reducing maternal mortality [1].

UN’s Maternal Mortality Estimation Inter-Agency Group reports that Suriname is one of the few countries with an increase in MMR from 127 in 1990 to 155 in 2015 [2, 3]. However, a confidential enquiry by Mungra et al. reported a MMR of 226 per 100.000 live births in 1991-1993, suggesting a 31% decrease instead of the 25% increase as suggested by the UN [4, 5]. However, it is unclear whether, and if so, to what extent, vital registration has become more reliable over the years.

Maternal health outcomes are strongly associated with higher capital levels, suggesting that an increase in Gross National Income (GNI) per capita should correspond with a reduction in maternal mortality [6]. Suriname was upgraded from lower-middle income country to upper-middle income country in 2013 as the GNI increased from $1430 in 1990 to $9370 in 2013 [7]. Yet, progress made on different basic health indicators (e.g. under five mortality, health insurance coverage and maternal mortality) in the country is relatively marginal [8].

According to WHO-estimates, Suriname (MMR 155) belongs to the four worst performing countries in Latin America and the Caribbean (Haiti - MMR 359, Guyana - MMR 229 and Bolivia - MMR 206) [1–3]. These are, in contrast to Suriname, low and lower-middle income countries. Suriname’s poor performance concerning maternal mortality is unexplained, as the country performs fairly well on maternal health indicators, e.g. skilled professionals attended 96% of the deliveries in the coastal area and 77% in the rural interior and antenatal care visits occurred at least once in 91% of the pregnant women and at least four times in 67% [8].

Therefore, the aim of the study is first to identify all maternal deaths in Suriname from 2010 to 2014, second to determine whether maternal deaths were accurately registered and classified, third to assess the reduction of maternal deaths in 25 years, fourth to perform an in-depth audit of the deaths and finally to determine the level of substandard care.

## Methods

### Study design

A reproductive age mortality survey (RAMoS) was conducted, using different methods to identify maternal deaths nationwide in Suriname between January 1st 2010 and December 31st 2014.

### Study setting

**Fig. 1:**
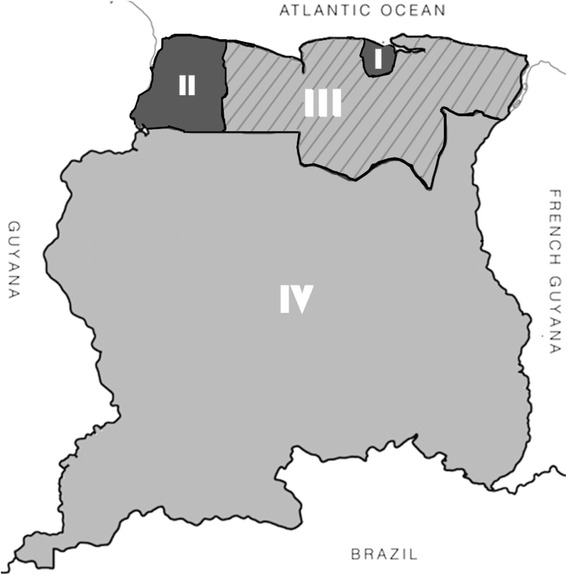
Map of Suriname divided in the urban (I-II), rural coastal (III) and rural interior (IV)

Suriname is a multi-ethnical South American country with a population of 541,638 served by four referral hospitals in the capital, Paramaribo, and one hospital near the western coast, Nickerie. In addition to general practitioners, Regional Health Services (RGD) and Medical Mission (MZ) are responsible for primary healthcare. RGD comprises of 43 facilities serving the whole coastal area and the Medical Mission has 56 health posts throughout the interior. Figure 1 demonstrates the urban area I (Paramaribo) and II (Nickerie), rural coastal area III and rural interior IV. Annually approximately 10,000 live births take place, of which hospitals cover an estimated 82% and primary health institutions 10%, 4% of deliveries are at home and the remaining 4% is unknown [9]. Social insurance, which is for the near poor and poor population, covers an estimated 45% ofthe general population. The ethnic distribution among the female population is Hindustani (28%), Maroon (24%), Creole (18%), Javanese (14%), Mixed (14%) and other (2%) [10].

### Classification & definitions

According to the ICD-MM a pregnancy-related death is the death of a woman while pregnant or within 42 days of termination of pregnancy, irrespective of the cause [11]. A maternal death is the death of a woman while pregnant or within 42 days of termination of pregnancy, irrespective of the duration and site of the pregnancy, from any cause related to or aggravated by the pregnancy or its management but not from accidental or incidental causes. Direct obstetric deaths are those resulting from obstetric complications, while indirect obstetric deaths are those resulting from either a previous existing disease or a disease that developed during pregnancy and which is not due to direct obstetric causes, but which is aggravated by physiologic effects of pregnancy. In unspecified maternal deaths the underlying cause is unknown or cannot be determined. Late maternal deaths are direct or indirect deaths, more than 42 days, but less than 1 year after termination of pregnancy. MMR is the number of direct, indirect and unspecified maternal deaths per 100,000 live births [11, 12].

#### Data collection

##### Vital registration

Maternal deaths in Suriname are identified mainly by the collection of death certificates and sporadic informing in the hospitals. No independent surveillance systems are adapted to investigate deaths in women of reproductive age. Notification of death is compulsory by law. However burial can take place without the official death certificate, when there is ‘an act of death’ (an unofficial note signed by a medical doctor). The death certificate is filled in afterwards and often received with a delay (>3 months) and in 15% not received at all. In addition the death certificate lacks a pregnancy checkbox [13]. Identified maternal deaths are not reviewed and thus not classified. Due to a lack of classification, most accidental/incidental deaths and late maternal deaths are also included in the official maternal mortality statistics.

##### Reproductive age mortality survey (RAMoS)

The RAMoS consisted of different steps. First, case records of maternal deaths from 2010 to 2014 identified by vital registration were collected. Second, all medical records of deceased women aged 10 to 50 years in our study period were collected from the archives of all hospitals and the primary health care institutions (Medical Mission and Regional Health Services). Third, The Central Bureau of Civil Affairs provided a list of all deceased women in the country between 2010 and 2014 with an offspring in the preceding year. Fourth, an inventory was performed in the largest mortuary (receiving also deaths occurring outside health care institutions). Fifth, obstetric health care professionals in all facilities were asked their knowledge on local maternal deaths in the past 5 years.

Medical records were collected and examined extensively and in case of an incomplete file involved health care professionals were interviewed. Verbal autopsy with family member(s) was performed when maternal deaths occurred outside of the hospital. This was conducted according to the WHO-instrument on verbal autopsy [14]. All available information was gathered (i.e. laboratory and pathology reports, in delivery-books and autopsy information).

An elaborate clinical case summary of every pregnancy-related death was made according to the FIGO-LOGIC *MDR: Clinical summary form* tool [15]. Information on patients, health care providers and hospitals was kept strictly confidential.

An expert committee, consisting of different obstetricians, an internal medicine specialist or anaesthesiologist and midwives, audited all pregnancy-related deaths with two authors (LK and KV) presenting and moderating the sessions. When no consensus was achieved, external expert opinion (JR and HK) was sought. The committee reviewed the cases and agreed to a mode of death, underlying cause, contributing factors and classified each death using *WHO guidelines on applications of ICD-MM* [12]*.* Substandard care factors were analysed according to an adapted version of the FIGO-LOGIC MDR *Grid analysis of clinical case management* form [15]. Due to lack of guidelines substandard care was defined as a deviation from ‘standard practice’ according to local clinicians.

#### Data analysis

Data were manually entered into IBM SPSS version 21.0 (Armonk, New York, USA) for analysis. All maternal deaths were individually analysed and cross-linked with registered maternal deaths by civil registration. Causes, contributing factors and substandard care factors were recoded into categorical variables.

## Results

**Fig. 2:**
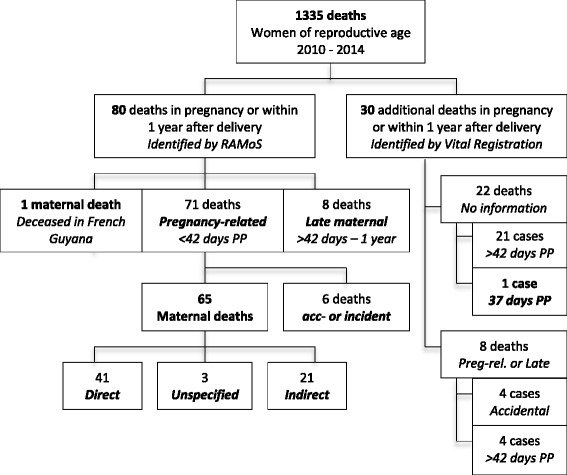
Flowchart of pregnancy related deaths in Suriname (RAMoS and vital registration)

Of the 1335 deceased women of reproductive age between 2010 and 2014, 71 were pregnancy-related and 65 were maternal deaths (Fig. 2). The 65 maternal deaths were identified among 50.051 live births, resulting in a MMR of 130 with an annual range from 69 to 154 per 100.000 live births (Table 1).

**Table 1:** MMR and number of maternal deaths found by RAMOS in comparison to vital registration

	2010	2011	2012	2013	2014	Total
Live births	9712	9703	10,217	10,012	10,407	50,051
RAMoS						
MMR	154	144	69	130	154	130
Maternal deaths	15	14	7	13	16	65
Vital registration						
MMR	82	103	49	120	125	96
Maternal deaths	8	10	5	12	13	48
Underreporting						
Misclassification of causes (%)	50	60	60	75	70	65
Misidentification (%)	47	28	30	8	18	26
Correction factor	1.88	1.40	1.40	1.08	1.23	1.35

### Underreporting

Underreporting occurred by misidentification in 26% (*n* = 17) and by misclassification in 65% (*n* = 31) (Table 1). The predictive value for the current vital registration to identify maternal deaths is 74% (48/48 + 17).

The maternal deaths not identified by vital registration (*n* = 17) occurred in the hospitals in 88% (*n* = 15) or at home in 12% (*n* = 2). The causes of these hospital-deaths were infectious diseases in 87% (*n* = 13), admitted and deceased on non-obstetric wards. These death certificates did not indicate or suggest that the woman was or had been pregnant. Maternal deaths, which were identified by vital registration but were classified incorrectly, consisted of deaths without the cause mentioned on the death certificate (*n* = 9), non-obstetric diseases (*n* = 13), deaths complicated with more than one diagnosis (*n* = 8) and cases in which the mode of death was reported on the death certificate rather than the underlying cause (*n* = 17).

Apart from the 48 true maternal deaths identified by vital registration, another 5 maternal deaths were incorrectly classified as maternal deaths (these were accidental or incidental causes or late maternal deaths).

### Characteristics of maternal deaths

The women in Suriname who died during pregnancy, childbirth or puerperium lived in a rural coastal area or in the rural interior in respectively 15 and 17% (Fig. 1 and Table 2). Maternal deaths, however, occurred in these areas in respectively 5 and 6%. Maternal deaths in urban hospitals (84%) occurred on the ICU (60%), ward (30%) or emergency or operating room (10%). Characteristics of maternal deaths are shown in Table 3.

**Table 2:** Demographics of Surinamese population in relation to maternal deaths

	Area I	Area II	Area III	Area IV
	Urban Paramaribo Wanica	Urban Nickerie	Rural coastal Coronie, Saramacca, Para, Commewijne, Marowijne	Rural interior Brokopondo Sipaliwini
General population, *n* = 534.189	66%	6%	18%	10%
Live births (2010-2014), *n* = 50.051				
Residency	67%	4%	18%	11%
Location of delivery^a^	77%	5%	5%	5%
Maternal deaths, *n* = 65 (%)				
Location of residence	40 (62)	4 (6)	10 (15)	17
Location of death				
Hospital, *n* = 55 (84)	53 (81)	2 (3)	N/A	N/A
Primary health care, *n* = 5 (8)	-	-	3 (5)	2 (3)
Home, *n* = 5 (8)	3 (5)	-		2 (3)
MMR per 100.000 live births	145	80	120	160

^a^Unknown location of live births in 8% (of which 50% of live births at home)

**Table 3:** Socio-demographic and obstetrical characteristics of all maternal deaths

	*n* = 65 (%)
Age	
< 20	11 (17)
20–35	42 (64)
36–50	12 (18)
Mean, range of age	29, 16-45
Ethnicity	
Hindu	12 (18)
Creole	13 (20)
Maroon	24 (37)
Javanese	8 (12)
Indigenous (Amerindians)	3 (5)
Mixed	5 (8)
Insurance (*n* = 63)	
Social insurance (poor)	45 (69)
State Health	10 (15)
Private	8 (12)
Antenatal care (*n* = 58)	
None	12 (23)
< 4	8 (15)
≥ 4	33 (62)
Parity at time of death (*n* = 62)	
0	5 (8)
1	14 (23)
2	17 (27)
≥ 3	26 (42)
Pregnancy state	
GA < 16 weeks	5 (8)
Antepartum	15 (23)
GA 16 – 27 weeks	4 (6)
GA > 28 weeks	9 (11)
Durante partum (GA > 37)	4 (6)
Post-partum	41 (63)
Mode of delivery (*n* = 41)	
Spontaneous, vaginal	25 (61)
Ventouse	3 (7)
Caesarean-section	13 (32)
Perinatal death (*n* = 57)	
Yes	36 (64)
Intrauterine fetal death	8 (14)
Post-partum (<7 days)	8 (14)

Social insurance, indicating the (near) poor, covered 69% (*n* = 45) of the deceased women. Socially insured women were maroons or creoles in 75% (*n* = 34) of the cases. Anaemia (Hb ≤ 6.0 mmol/L) complicated 45% of the cases. Post-mortem investigation was performed in 3% (*n* = 2) of maternal deaths.

### Classification and causes of maternal deaths

**Fig. 3:**
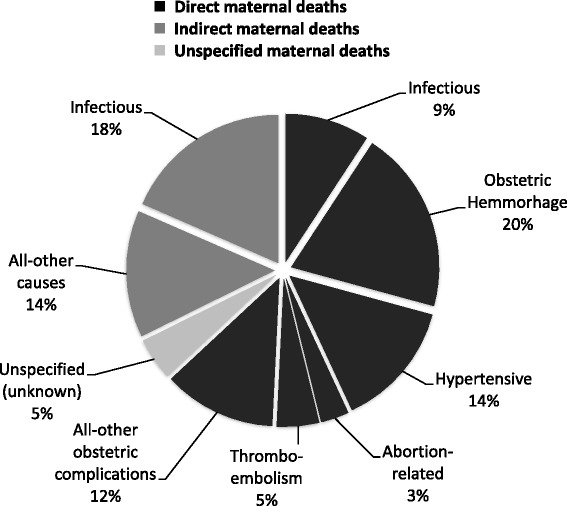
Classification of underlying causes of the maternal deaths (*n* = 65)

Of the 65 maternal deaths, 41 (63%) were due to direct causes, 21 (32%) due to indirect causes and three (5%) maternal deaths were classified as unspecified because the cause of death was unknown (Fig. 3). The two leading causes of maternal mortality were obstetric and non-obstetric sepsis (*n* = 18, 27%) and obstetric haemorrhage (*n* = 13, 20%).

Obstetric haemorrhage was mainly due to postpartum haemorrhage (*n* = 11, 85%) caused by uterine atony (29%), retained placenta (23%), ruptured uterus (15%), vaginal / cervical tear (8%), and unspecified causes (10%). Underlying cause of all ante partum haemorrhages was placental abruption (*n* = 2, 15%).

Hypertensive disorders and its complications (e.g. cerebral bleeding, HELLP, eclampsia) accounted for 14% of maternal deaths. However, hypertensive disorders, such as pregnancy induced hypertension and pre-eclampsia, were diagnosed in 30% of all maternal deaths. Though not the underlying cause of death, they were commonly classified as a contributing factor.

The remaining other causes of direct maternal deaths (*n* = 8, 12%) were four probable amniotic fluid embolisms, one obstructed labour, one suicide by intoxication at 24 weeks, one case of acute fatty liver of pregnancy with consequently hepatic encephalopathy and multi-organ failure. The underlying cause of one case remained unknown as the woman died without any reported symptoms within a few hours after caesarean section for foetal indication.

Sepsis occurred either due to direct obstetric complications (9%) of which one third had puerperal sepsis while being HIV positive or due to medical conditions aggravated by the pregnancy (e.g. non-obstetric septicaemia, pneumonia, gastro-enteritis, AIDS) and therefore were classified as indirect maternal deaths (18%).

The other non-sepsis indirect maternal deaths (*n* = 9) concerned two cases of endocarditis resulting in heart failure, one pulmonary bleeding caused by idiopathic thrombocytopenia, one case of end-stage renal failure due to diabetes and one woman, diagnosed with pre-existent hypertension, died due to a cerebrovascular accident.

Substandard care factors were found in 95% (*n* = 56/59) of the cases (Table 4). More than 5 substandard care factors were present in 55% of cases.

**Table 4:** Substandard care factors analysed in 59 of the 65 cases of maternal deaths

	*n* = 59 (%)
Professional factors	
Quality	47 (80)
Availability	11 (19)
Attitude / work-ethic	13 (22)
Medical service factors	
Wrong or delay in diagnosis	35 (59)
No/inadequate treatment	46 (78)
Poor monitoring	35 (59)
Communication	19 (32)
Unavailability	
Diagnostics (eg. lab, pathology)	8 (14)
ICU-bed (*n* = 45)	11 (24)
Blood (*n* = 31^*^)	10 (32)
Supplies / medication	12 (20)
Patient factors	
Poor compliance to treatment	13 (22)
Refusing treatment	4 (7)
Delay in transportation	9 (15)

In 80% of the cases care provided by health professionals was below the standard due to delay in diagnosis (59%), inadequate treatment (78%) or poor monitoring (59%). Blood transfusion was unavailable in 10 of 31 cases (32%) when this was required. An ICU bed was not available when requested in 11 (24%) of 45 cases. The committee agreed that in 47% of the maternal deaths substandard care factors certainly (21%) or most likely (26%) led to death.

## Discussion

The MMR in Suriname is 130 per 100,000 live births between 2010 and 2014. Mungra et al. reported a MMR of 226 between 1991 and 1993, which indicates a 42% reduction in maternal deaths and an improvement in underreporting from 64% to 26% [4, 5]. A comparison of the MMR and underreporting is difficult, as to our best knowledge there are few countries that have performed a RAMoS of confidential enquiry [16–19].

### Underreporting

Though our study suggests that, over the years, there is a growing reliability on identification of maternal deaths, the underreporting rate in Suriname (26%) is still higher than reported in Jamaica (20%), Argentina (9.5%) and Mexico (13%) [16–19].

The underreporting due to misidentification of maternal deaths in Suriname can be explained by numerous facts: first, physicians are not obliged to report maternal deaths. Second, part of the death certificate (including the cause of death) is not always available as it is not obliged to be completed before the burial takes place. Third, the death certificate does not include a pregnancy checkbox and finally no active enquiry or RAMoS is performed. The effectiveness of a pregnancy check box on death certificates has proven to be effective in identifying pregnancy-associated mortality [20, 21]. Misclassification of deaths by vital registration in Suriname can be explained by different factors. First, maternal death causes are designated by the ICD-code on the death certificate (patient records frequently unavailable), while the ICD-MM coding alone is considered inadequate [22]. Second, post-mortem investigations are rare. Third, verbal autopsies and maternal death reviews are not performed to identify causes. These last strategies are best in identifying causes and evaluating quality of care in order to improve [11, 14, 15, 22].

### Characteristics & causes

Social insurance as a marker indicating poverty was found in the majority of maternal deaths (69%), while less than half of the general population had social insurance. A difference in ethnicity is seen between the general female population (Hindustani 28%; Maroon 24%; Mixed 14%) and the maternal deaths (Hindustani 18%; Maroon 37%; Mixed 8%). Similar to two decades ago, obstetric haemorrhage is the most common direct cause of death, which is lower than reported in low-income countries (27%) and higher than in high-income countries (16%) [3]. Hypertensive disorders are known to be an important cause of maternal deaths in Latin America and the Caribbean (22%) [1–3]. While eclampsia was the underlying cause in just 14% of the deaths in Suriname, it was an important contributing factor (30%) to deaths with another underlying cause. The authors advise health authorities to implement nationwide protocols for the prevention and management of hypertensive disorders and post-partum haemorrhage. Illegal abortion is the cause of death in only one case (1.5%), which is in great contrast to the 12% abortion-related deaths in Latin America and the Caribbean [3, 17, 19]. Although illegal in Suriname, most abortions are self-induced with misoprostol and women present with an incomplete abortion after which safe surgical evacuation is performed in the hospital by a gynaecologist or gynaecologist in training. However, since termination of pregnancy is not registered, underreporting could have occurred.

Indirect maternal deaths (32%), in particular non-obstetric sepsis (18%), accounted for a greater part of the maternal deaths in our study compared to the 27% of other Latin American and Caribbean countries [3]. Therefore, we recommend that these maternal deaths from should be analysed in detail to gain more knowledge of underlying causes, circumstances and preventive measurements.

### Why do pregnant women die in Suriname and what can we do about it?

The most striking finding of our survey is that the majority of maternal deaths occurred in hospital (85%) with the most important substandard care factor being delay in diagnosis (59%) and delay in treatment by health care providers, and less frequently due to patient delay (15%). This finding necessitates actions such as training and retaining skilled staff and implementation of evidence-based guidelines. Another important finding is that most of the deaths occurred postpartum, indicating that improvements can be made in the care provided in the period after birth. Patients should be provided with more information. We advise more frequent and qualitative better postnatal checks and if necessary home-visits should be performed. Lastly, a great number of maternal deaths occurred on the wards (30%) and the monitoring of patients is found to be inadequate in 59% of these cases. Implementation of an early warning system for timely interventions in order to reduce serious adverse events has been proven effective and is recommended [23].

Limitations of this study are indwelled in its retrospective nature. Though we performed a robust enquiry, maternal deaths could have been missed, especially if they occurred outside of health care facilities or during early pregnancy. In addition, not all case files were available, records were often incomplete and post-mortem investigations were generally not performed. This affected the quality of the classification of causes and evaluation of substandard care during the maternal death reviews. Finally, but most importantly, due to lack of national data on characteristics of the pregnant population, pregnancy and delivery, we were not able to perform multivariate analysis and assess risk factors.

## Conclusion

Suriname has a high MMR compared to other Latin American countries and the Caribbean with similar or lower income economies. We highly recommend (1) to improve national data surveillance, (2) install a maternal mortality committee to review all maternal deaths, (3) implement an early warning score and national guidelines on postpartum haemorrhage and eclampsia and (4) improve postnatal care strategies. Lastly, as maternal mortality is merely the tip of the iceberg, severe morbidity research should be conducted to assess and prevent severe obstetric complications and make progress to reach the SDG of a MMR <70 in 2030.

## Abbreviations

BOG: Bureau of public health (Bureau Openbare Gezondheidszorg)
FIGO-LOGIC: International federation of gynaecology and obstetrics – leaderschip in obstetrics and gynaecology for impact & change
GNI: Gross national income
ICD-MM: International statistical classification of diseases – maternal mortality
ICU: Intensive care unit
MDG: Millennium development goals
MDR: Maternal death review
MMR: Maternal mortality ratio
MZ: Medical Mission (Medische Zending)
RAMoS: Reproductive age mortality survey
RGD: Regional health services (Regionale Gezondheidsdienst)
SDG: Sustainable development goals
